# Diurnal variation of serum phosphorus concentrations in intact male adult domestic cats

**DOI:** 10.1111/jvim.17202

**Published:** 2024-09-26

**Authors:** Chih‐Fan Chiang, Raphael Vanderstichel, Jonathan Stockman, Jennifer A. Larsen, Andrea J. Fascetti

**Affiliations:** ^1^ Veterinary Medical Teaching Hospital, School of Veterinary Medicine University of California, Davis Davis California USA; ^2^ Department of Clinical Veterinary Sciences, College of Veterinary Medicine Long Island University Brooklyn New York USA; ^3^ Department of Molecular Biosciences, School of Veterinary Medicine University of California, Davis Davis California USA

**Keywords:** calcidiol, calcium, circadian rhythm, feline, PTH

## Abstract

**Background:**

Monitoring serum phosphorus concentrations is crucial in the management of chronic kidney disease in cats. The diurnal variation of serum phosphorus concentration may affect clinical assessment.

**Hypothesis/Objectives:**

Characterize the diurnal variation of serum phosphorus concentration in cats and determine the associations between changes in serum phosphorus concentration and several metabolites of phosphorus metabolism.

**Animals:**

Six apparently healthy, intact male, specific‐pathogen‐free cats were housed in a photoperiod, humidity, and temperature‐controlled facility.

**Methods:**

Blood sampling was performed hourly for 24 hours to obtain the serum concentrations of phosphorus, ionized calcium, parathyroid hormone, and calcidiol. Results were analyzed using linear mixed‐effect models to determine the significance of diurnal variation and associations between serum phosphorus concentrations and other metabolites over time.

**Results:**

Diurnal variation in serum phosphorus concentration was noted with an apex around 11:00 followed by gradually declining concentrations to reach the nadir around 23:00. The serum phosphorus concentration again increased through the early morning on the next day. An approximately 25% difference in serum phosphorus concentration at the apex and the nadir was documented. A non‐linear relationship between the serum concentrations of phosphorus and ionized calcium over time was identified.

**Conclusions and Clinical Importance:**

Diurnal variations of serum phosphorus concentration and associations between the trending of serum phosphorus and ionized calcium concentrations were evident in a group of clinically healthy adult cats housed in a controlled environment. These findings can help in the interpretation of clinical and research data regarding calcium and phosphorus metabolism and kidney health in cats.

**Abbreviations**
AAFCO: Association of American Feed Control Officials
AR: autoregressive
BCS: body condition score
Ca: calcium
Ca:P: calcium to phosphorus ratio
CKD: chronic kidney disease
FGF 23: fibroblast growth factor 23
ICC: intraclass correlation coefficients
IQR: interquartile range
IRIS: International Renal Interest Society
MA: moving average
Me: median
NRC: National Research Council
P: phosphorus
PTH: parathyroid hormone
SDMA: symmetric dimethylarginine

## 1 INTRODUCTION

Cats with chronic kidney disease (CKD) suffer from clinical signs such as lethargy and hyporexia, which can negatively impact quality of life and longevity. A median survival time of 4 years was reported in cats with progressive CKD.^1^ Hyperphosphatemia can be seen in cats with CKD because of decreased renal excretion. Regardless of its variable severity and prevalence depending on the stage of CKD, hyperphosphatemia is known to contribute to oxidative damage to nephrons, soft tissue mineralization, and progression of CKD.^2^, ^3^ Currently, monitoring serum phosphorus (P) concentration and managing hyperphosphatemia using dietary and medical interventions are recommended in cats with CKD.^4^ Blood P concentrations in cats are known to be influenced by hormones (eg, parathyroid hormone [PTH]) and diet (eg, amount and type of dietary P), whereas diurnal variations (or circadian rhythm) of blood P concentrations have been described in humans.^5^, ^6^, ^7^, ^8^, ^9^ Overall, the rhythm of blood P concentration in humans was characterized by an apex or plateau after midnight (00:00) followed by a nadir before noon (12:00); concentrations between apex and nadir differed by approximately 30%.^7^, ^8^, ^9^ If such variation exists in cats, it could undermine the consistency of clinical assessments and confound ongoing research investigating the impact of P on kidney function in cats. However, little information is available on whether or not diurnal variation of blood P concentration exists in cats and whether such variation can be attributed to photoperiod, hormonal activities, and dietary intake in cats.

One study assessed plasma P concentration in intact male cats after the consumption of diets formulated to be nutritionally adequate.^10^ In that study, postprandial increases in plasma P concentrations were noted 1 hour after feeding and the increase in plasma P concentration persisted for 8 hours, but additional timepoints and pertinent hormones were not assessed in that study.^10^ Our objective was to characterize the diurnal variation of serum P concentration in apparently healthy cats housed in a temperature, humidity, and photoperiod‐controlled environment and to determine its relationship with changes in the concentrations of serum ionized calcium (ionized Ca), calcidiol, and PTH. We hypothesized that diurnal variation in serum P concentrations in cats would be similar to that observed in humans, and that the variation could not be fully attributed to the metabolites mentioned above.

## 2 MATERIALS AND METHODS

### 2.1 Animals

Eight intact male, specific‐pathogen‐free, domestic short‐haired cats (between 1 and 5 years old) housed at the Feline Nutrition and Pet Care Center at the University of California (Davis, California) were identified on Day −17. The cats were routinely assessed by veterinary technicians and board‐certified laboratory animal veterinarians, and their medical histories and physical examinations were unremarkable. All cats were assessed as having an ideal body condition score (BCS)^11^ and normal muscle condition.^12^

The facility was temperature, humidity, and photoperiod controlled (lights on for 14 hours from 06:00 to 20:00 and off for 10 hours daily). The cats had free access to clean drinking water and a commercial dry cat food formulated for all life stages according to the Association of American Feed Control Officials (AAFCO).^13^

### 2.2 Diet analysis

A sample of the diet was submitted to a reference laboratory (Eurofins Nutrition Analysis Center, Des Moines, Iowa) for proximate analysis of crude protein, crude fat, crude fiber, moisture, ash, P, Ca, sodium, potassium, and calcitriol using validated analytical methods, including those defined by the Association of Official Agricultural Chemists. A sample of the diet was also submitted to the Amino Acid Laboratory at the University of California (Davis, California) for determination of soluble P concentration following analytical methods described elsewhere.^14^ Samples were processed in distilled deionized water and hydrogen chloride for 2 and 90 minutes. The fraction of soluble P in the diet was expressed as the ratio of the soluble P concentration (total orthophosphate; the mean of sample duplicates) to the total P concentration (results of proximate analysis).

### 2.3 Experimental design

The protocol for this prospective cohort study was approved by the Institutional Animal Care and Use Committee of the University of California (Davis, California). All cats underwent laboratory analysis on Day −15, including serum biochemistry panel, CBC, urinalysis, total T4 concentration (performed at the Veterinary Clinical Diagnostic Laboratories at the University of California, Davis, California), symmetric dimethylarginine (SDMA; IDEXX Reference Laboratories, Westbrook, Maine), and vitamin D profile (ionized Ca, PTH, and calcidiol; Veterinary Diagnostic Laboratory at Michigan State University, East Lansing, Michigan).

Starting Day −14, cats were acclimated to a twice‐daily feeding regimen; the amounts offered were based on the calculated energy requirement. Meals were offered at 08:00 and 18:00 for a 1‐hour period, and food consumption was measured and recorded. Daily calorie intake was adjusted as needed to maintain each cat's body weight, muscle mass, and ideal BCS.

On the morning of Day −1, each cat was sedated to facilitate placement of an indwelling IV catheter into the jugular vein by a board‐certified specialist in veterinary emergency and critical care. The catheter was infused with heparinized saline to maintain patency until 08:00 on Day 0. After catheter placement, each cat was transferred to individual housing and monitored until full recovery from sedation. Elizabethan collars were placed to avoid the removal of the catheters.

On Day 0 at 08:00, hourly blood sampling was initiated, utilizing a push‐pull technique described elsewhere.^15^ At each sampling time point, 2 mL of whole blood was collected for further processing and analysis of serum P, ionized Ca, PTH, and calcidiol concentrations. At 09:00, all cats were offered their typical meal (providing 50% of their daily intake). No additional food was offered until the conclusion of serial blood sampling at 07:00 on Day 1 to avoid interference of dietary P on serum P concentration and to avoid excessive stress to the cats by prolonged fasting.

### 2.4 Sample processing and analysis

At each time point, whole blood samples were processed immediately into separate serum and plasma fractions, which then were stored short‐term at −20°C. On Day 1, serum samples for P analysis were submitted to the Veterinary Clinical Diagnostic Laboratories at the University of California (Davis, California). Serum samples for ionized Ca, PTH, and calcidiol analyses were shipped overnight on dry ice to the Veterinary Diagnostic Laboratory at Michigan State University (East Lansing, Michigan), and were analyzed using validated assays for both research and clinical diagnosis purposes on a pay‐for‐service basis. Serum ionized Ca concentration was determined using a commercially available analyzer and ion‐specific electrodes (Nova Biomedical, Waltham, Massachusetts), PTH was quantitated using a commercially available automated chemiluminescent immunoassay kit (Immunodiagnostic Systems, East Boldon, the United Kingdom), and calcidiol was determined using a commercially available radioimmunoassay kit (Immunodiagnostic Systems, East Boldon, the United Kingdom). Plasma samples were stored at −80°C for possible additional analyses.

### 2.5 Statistical analysis

Descriptive statistics were used to summarize baseline concentrations of variables (serum P, ionized Ca, PTH, and calcidiol) monitored in the 24‐hour serial blood sampling period in participating cats. Median (Me) and interquartile range (IQR; defined as the range between the 1st and 3rd quartiles) were used along with boxplots to provide an overview of data distribution.

Linear mixed‐effect models were used to predict serum P, ionized Ca, PTH, and calcidiol concentration changes in the 24‐hour serial blood sampling period in participating cats. In the statistical models, cats were included as a random effect, and a first‐order autoregressive model was imposed on the residuals given the temporal within‐cat autocorrelation nature of the repeated measurements. Models were fit with the restricted maximum likelihood estimation method unless nested models were compared. Various autocorrelation models (namely independent, autoregressive [AR(1), AR(2)], moving average [MA(1), MA(2)]) were compared against each other using likelihood ratio tests or Akaike's information criterion, as appropriate.^16^, ^17^ The 24‐hour concentration changes of each metabolite (serum P, ionized Ca, PTH, and calcidiol) were modeled as 2 separate fixed effect terms: 1 as a sine function [sine([h/24] × 360) (π/180)] and the other as a cosine function [cosine([h/24] × 360) (π/180)] to assess the significance of diurnal variation.^17^ Non‐linearity of the remaining fixed effects (the concentrations of serum ionized Ca, PTH, and calcidiol) was assessed using polynomial terms (up to a cubic transformation) by centering the first term and applying the squared and cubed functions to the second and third term, respectively.

Intraclass correlation coefficients (ICC), producing correlation estimates for any 2 observations taken within a cat in a 24‐hour period, were calculated for each serum variable using simplified linear mixed‐effect models, which included only cats as random effects, and no other fixed effects or assumed autocorrelation structures to the residuals.

Statistical model diagnostics were performed to ensure statistical assumptions were not violated, and included inspection of residuals and best linear unbiased predictions for the random effects for normality and homoscedasticity.^16^ All analyses were carried out using Stata (version v.17.0; StataCorp, College Station, Texas) and statistical significance was set at *P* < .05.

## 3 RESULTS

Two cats were removed from the study because of acute gastrointestinal signs or temperament not amenable to serial sampling. All remaining 6 cats ate the diet consistently from Day −14 to 0 and maintained ideal BCS and muscle condition despite minor weight change (median loss of 0.5% per week), and fully consumed the morning meal providing 50% of their daily intake on Day 0. Laboratory analysis of the diet indicated that all assessed nutrients exceeded the nutrient guidelines for adult cats as established by the AAFCO and the National Research Council (NRC; Table 1).^18^ The P solubility was similar between samples processed in both distilled deionized water and hydrogen chloride for 2 minutes (25% and 28% of the measured total P, respectively), but was higher in samples incubated in distilled deionized water and hydrogen chloride for 90 minutes (54% and 57%, respectively).

**TABLE 1:** Concentrations of key nutrients in the feline diet^a^ offered in the present study and comparison to AAFCO^13^ and NRC^17^ values.

Parameters	Unit	As fed basis	Per 1000 kcal	AAFCO minimum concentration for adult cat diets (per 1000 kcal)	NRC recommended allowance for adult cats (per 1000 kcal)
Calorie, calculated	kcal	378.7	‐	‐	‐
Crude protein	gram	34.06	89.94	65	50
Crude fat	gram	14.75	38.95	22.5	22.5
Crude fiber	gram	1.3	3.4	‐	‐
Moisture	gram	5.5	‐	‐	‐
Ash	gram	6.07	‐	‐	‐
NFE, calculated	gram	38.32	101.19	‐	‐
Calcium	gram	1.28	3.38	1.5	0.72
Phosphorus	gram	1.12	2.96	1.25	0.64
Ca:P	‐	1.1:1	1.1:1	‐	‐
Sodium	gram	0.28	0.74	0.5	0.17
Potassium	gram	0.68	1.79	1.5	1.3
Cholecalciferol	IU	144	380	70	70

*Note*: The sample was processed and the result was reported by Eurofins Nutrition Analysis Center, Des Moines, IA.

Abbreviations: AAFCO, Association of American Feed Control Officials 2023; NRC, National Research Council 2006; Ca:P, calcium to phosphorus ratio; NFE, nitrogen‐free extract.

^a^ Purina Cat Chow Complete dry is formulated to meet the nutritional levels established by the AAFCO Cat Food Nutrient Profiles for all life stages. Ingredients: chicken by‐product meal, ground yellow corn, corn gluten meal, whole grain wheat, rice, soy flour, beef fat preserved with mixed‐tocopherols, chicken, fish meal, liver flavor, phosphoric acid, calcium carbonate, salt, potassium chloride, choline chloride, minerals (zinc sulfate, ferrous sulfate, manganese sulfate, copper sulfate, calcium iodate, sodium selenite), taurine, vitamins (vitamin E supplement, niacin [vitamin B‐3], vitamin A supplement, calcium pantothenate [vitamin B‐5], thiamine mononitrate [vitamin B‐1], riboflavin supplement [vitamin B‐2], vitamin B‐12 supplement, pyridoxine hydrochloride [vitamin B‐6], folic acid [vitamin B‐9], vitamin D‐3 supplement, biotin [vitamin B‐7], menadione sodium bisulfite complex [vitamin K]), DL‐Methionine, Red 40, Yellow 5, Blue 2.

### 3.1 Baseline laboratory results

Baseline blood tests performed on Day −15 are tabulated in Table S1. Participating cats' anion gap (Me, 22 mmol/L; IQR, 20‐26) and serum urea nitrogen (Me, 25 mg/dL; IQR, 22‐27), creatinine (Me, 1.3 mg/dL; IQR, 1.1‐1.5), sodium (Me, 153 mmol/L; IQR, 151‐155), potassium (Me, 4.3 mmol/L; IQR, 4.2‐4.3), total Ca (Me, 9.8 mg/dL; IQR, 9.6‐10.0), ionized Ca (Me, 1.2 mmol/L; IQR, 1.2‐1.3), and P (Me, 4.7 mg/dL; IQR, 4.0‐5.1) concentrations were within the diagnostic laboratory's reference ranges. Serum albumin concentration was also within the reference range in all cats (Me, 3.9 g/dL; IQR, 3.7‐4.1), but total hypoproteinemia at 6.0, 6.1, and 6.1 g/dL (reference range, 6.6‐8.4) was noted in 3 cats because of hypoglobulinemia (2.3, 2.4, and 2.5 g/dL, respectively; reference range, 2.8‐5.4). Serum PTH concentration was above the reference range in 3 cats (4.1, 4.9, and 6.6 pmol/L, respectively; reference range, 0.7‐3.4), and serum calcidiol concentration was below the reference range in 5 cats (95, 102, 115, 121, and 125 nmol/L, respectively; reference range, 127‐335). In addition, SDMA concentration was above the reference range in 3 cats (15, 16, and 17 mcg/dL, respectively; reference range, 0‐14). Urine could not be obtained from 1 of the 3 cats with increased SDMA concentration at the time of blood sampling on Day −15; the remaining 5 cats had concentrated urine (urine specific gravity, 1.041‐1.052; Table S1).

### 3.2 24‐hour serial blood sampling on Day 0

**FIGURE 1:**
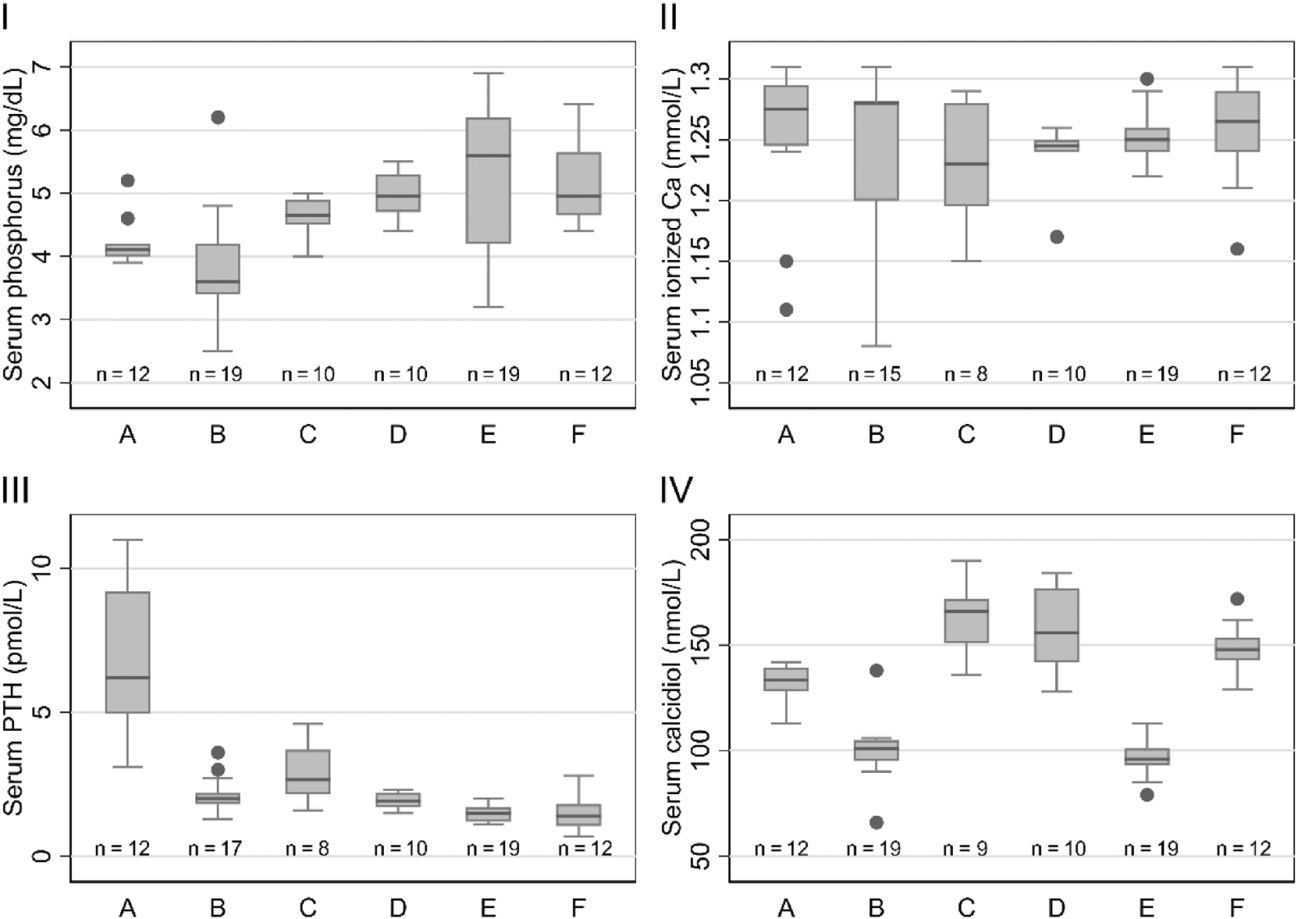
Boxplots showing the concentrations of serum phosphorus (I), ionized calcium (Ca; II), parathyroid hormone (PTH; III), and calcidiol (IV) in each cat throughout the 24‐hour serial blood sampling.

Because of technical challenges with catheter sampling and the fact that not all cats remained fully cooperative at all time points, adjustments were necessary to the planned sampling frequency, collection volumes, or both. As such, not all variables were measured in all cats at all time points. The concentrations of serum P, ionized Ca, PTH, and calcidiol throughout the 24‐hour blood sampling are summarized in Figure 1 as boxplots and in Tables S2–S5.

**FIGURE 2:**
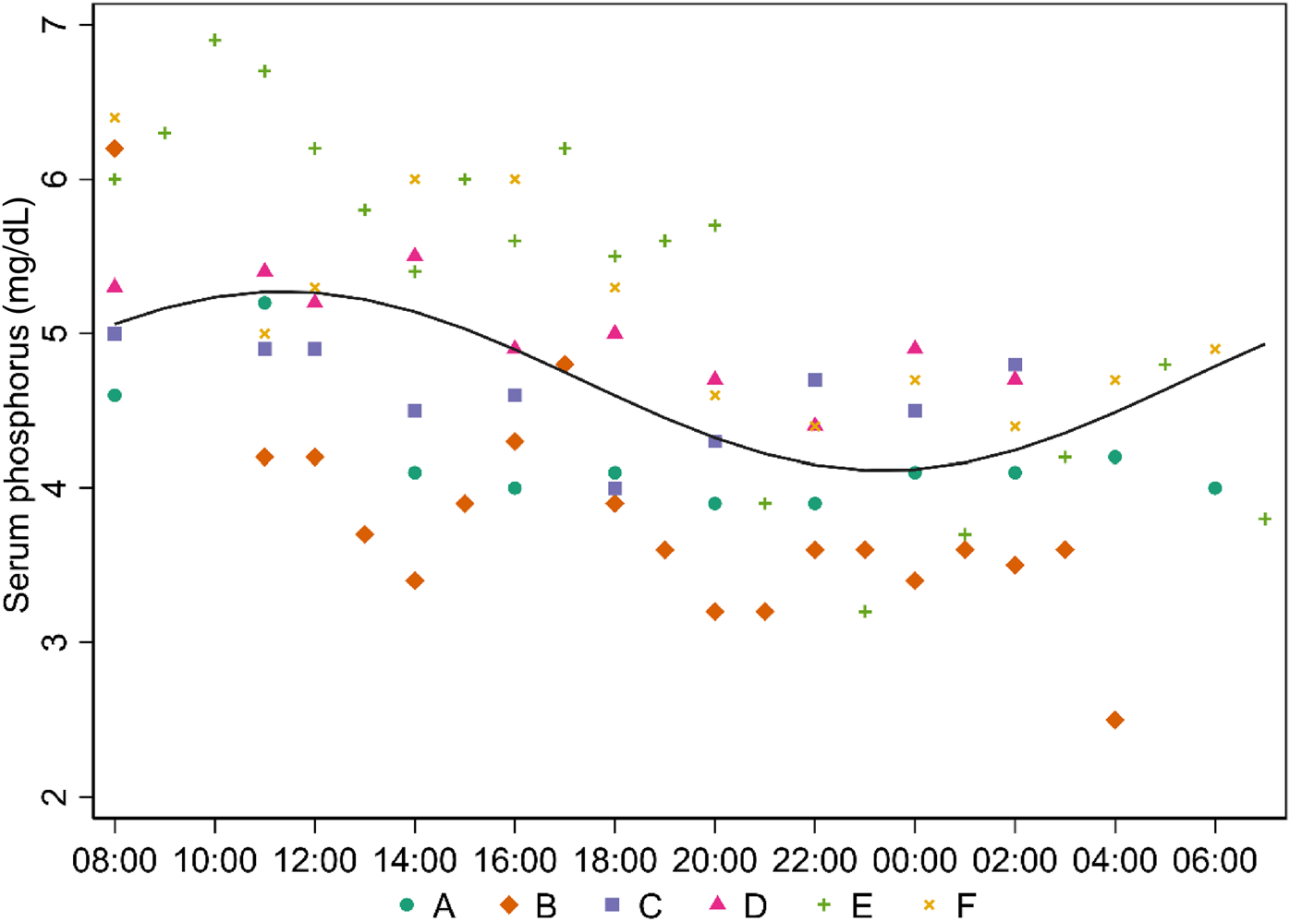
Serum phosphorus concentrations (n = 82) obtained during 24‐hour serial blood sampling in 6 cats (A, B, C, D, E, and F), starting at 08:00. The cats' normal meal was offered at 09:00; no other food was provided. The diurnal variation of serum phosphorus concentrations was modeled with a linear mixed‐effect model using sine and cosine functions, with cats as a random effect and a second‐order moving‐average structure was imposed on the residuals.

The 24‐hour concentration changes of serum metabolites were modeled using sine and cosine functions in linear mixed‐effect regression models, and the sine and cosine functions for serum Ca (*P* = .54 and .55, respectively), PTH (*P* = .55 and .25, respectively), and calcidiol (*P* = .88 and .10, respectively) failed to predict the diurnal variation of these serum metabolites throughout the 24‐hour serial blood sampling. Despite the effect of the sine function being insignificant in predicting serum P concentration changes (*P* = .50), the cosine function had a significant effect in predicting concentration changes of serum P in the present study (*P* < .001). The diurnal variation of serum P concentration is depicted in Figure 2, where the concentration apex was reached around 11:00 and the concentration gradually decreased to a nadir around 23:00, with an approximately 25% difference in serum P concentrations at the apex and nadir (Figure 2). Despite nonsignificant diurnal variation, the concentration changes of ionized Ca, calcidiol, and PTH also are depicted in Figure S1.

**FIGURE 3:**
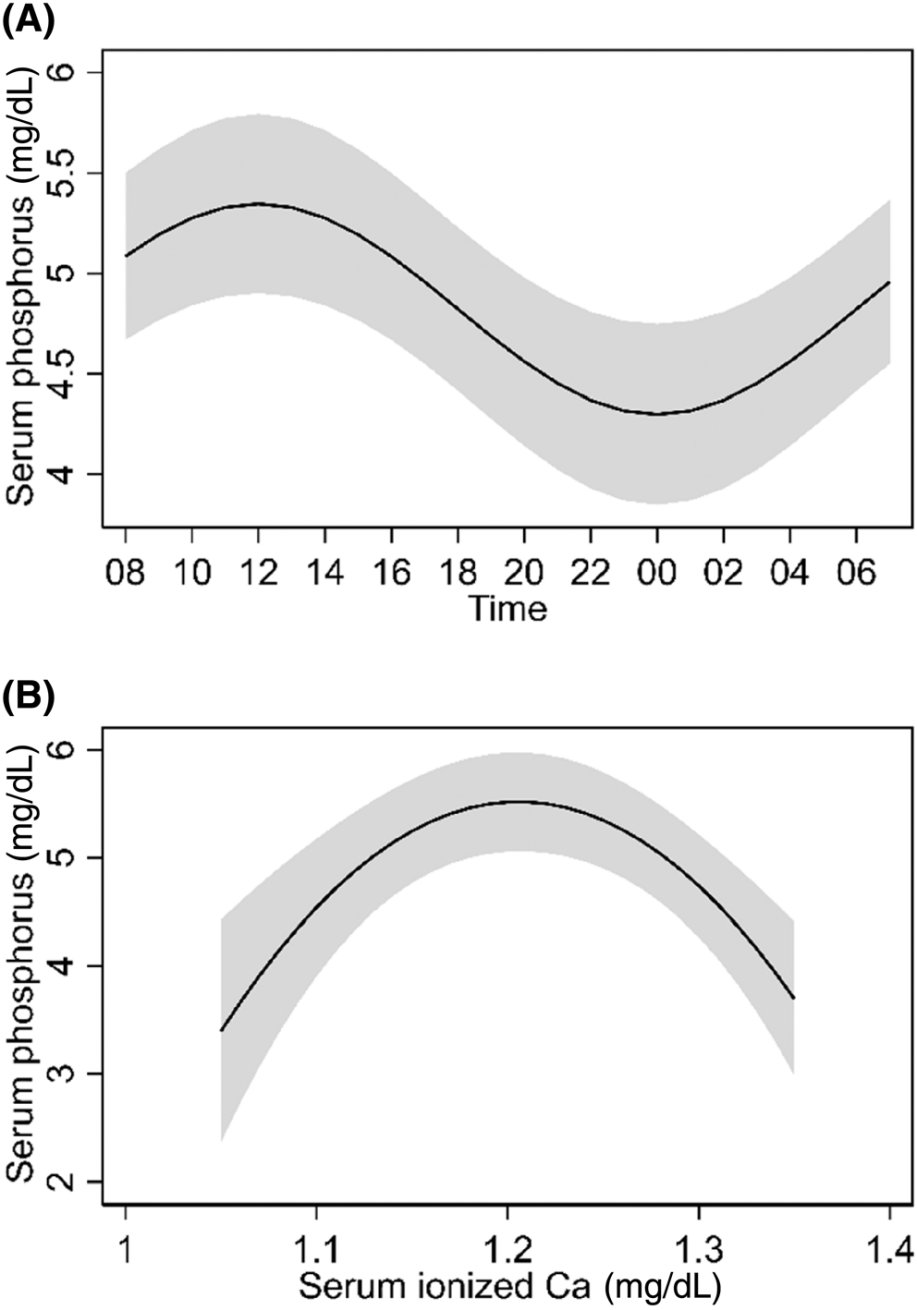
Predicted serum phosphorus concentrations in cats undergoing the 24‐hour serial blood sampling across time (A) and various ionized calcium (Ca) levels (B). Estimates were derived from a linear mixed‐effect model with sine and cosine functions for time (hours) and a polynomial function (squared term) for ionized Ca, with time fixed at noon for the ionized Ca predictors and Ca fixed at the median concentration of 1.25 mg/dL for the temporal predictors. A second‐order moving‐average correlation structure was imposed on the residuals (n = 76), and cats (n = 6; Cat A, B, C, D, E, and F) were included as a random effect. The shaded area represents the 95% confidence interval.

The diurnal variation of serum ionized Ca concentration was not significant. However, serum ionized Ca concentration was a significant predictor for serum P concentrations when modeled as a polynomial function (included in the regression model as a centered, first term [(Ca × 10)‐12.5] and a squared, second term [(Ca × 10)^2^]; *P* < .001) for both polynomial terms (Figure 3B). Figure 3B shows the marginal estimates from the final model for serum P concentrations with both time (hour, modeled as a cosine function) and serum ionized Ca concentration (mg/dL, squared polynomial function) as predictors in the model, along with random effects for cats and a second‐order MA autocorrelation structure imposed on the residuals. The estimated autocorrelations (rho) for the MA (2) structure were 0.459 and 0.379 for a 1‐ and 2‐hour lagged difference, respectively.

With a higher ICC, the concentrations of serum PTH (0.75) and calcidiol (0.84) concentrations for individual cats were less variable throughout the day compared with the concentrations of serum P and ionized Ca with ICC of 0.44 and <0.01, respectively. In other words, the serum PTH and calcidiol concentrations for individual cats were more stable during the 24‐hour blood sampling compared with the serum concentrations of P and ionized Ca despite the variability among cats (Figure 1).

## 4 DISCUSSION

Phosphorus plays a crucial role in the pathology of kidney diseases in cats.^2^, ^3^, ^4^ According to current guidelines published by the International Renal Interest Society (IRIS), the maintenance of serum P concentration between 2.7 and 4.6 mg/dL using dietary and medical intervention minimizes kidney disease progression in cats with IRIS CKD stages 2‐4.^4^ The diurnal variation (or circadian rhythm) of serum P concentration in cats documented in our study could negatively affect the consistency of clinical assessment and monitoring of cats with CKD.

In our study, the diurnal variations of serum P concentrations in cats were not consistent with those reported in humans.^5^, ^6^, ^7^, ^8^, ^9^ The circadian rhythm of blood P concentration in humans generally consisted of an apex or plateau between midnight (00:00) and early morning (around 04:00) and a subsequent gradual decrease to the nadir between 08:00 and 12:00.^5^, ^6^, ^7^, ^9^ Differences in the diurnal variation between humans and cats also were reported in blood pressure. Systolic blood pressure increased at both 08:00 and 19:00 in apparently healthy cats.^19^ Conversely, the blood pressure of humans decreased in late afternoon and then reached a nadir in the evening or by midnight.^20^ Interestingly, as another companion animal species, diurnal variation of plasma P concentration in dogs seemed to be similar to that reported in humans.^21^

Many fundamental differences between cats and humans likely contribute to the differences documented. Although humans and dogs are diurnal animals,^22^ cats are considered crepuscular to nocturnal animals.^23^ Other nocturnal animals, such as rats (apex around 12:00 and nadir around 24:00)^24^ and mice (apex around 12:00 and nadir around 20:00),^25^ share similar diurnal variations in blood P concentration with cats.

Circadian rhythms are modulated by clock genes in both transcriptional and posttranslational manners after the signals from the suprachiasmatic nucleus stimulated by the photoperiod cycle.^26^ Considering the pattern of photoperiodic stimulations throughout the day, the habitual behavior in 1 species could drive the pattern of its exclusive rhythm. Given the role the photoperiod cycle plays in circadian rhythm,^27^ our study was deliberately designed to follow the general day and night cycle used in other studies to facilitate comparisons to other species and to align with clinical settings.

The timing of meals also can modify the rhythm of serum P concentration. Although studies assessing circadian rhythms in humans commonly were designed to include meals offered around 08:00, 12:00, and 18:00,^5^, ^6^, ^7^, ^8^, ^9^ a purposefully designed study documented the impact of meal timing on the diurnal variation of serum P concentration in humans.^28^ In that study, 2 groups of healthy adult males were offered 3 meals with identical ingredients and nutritional profiles at the same time during the 24‐hour serial blood sampling except for the timing of dinner (early or late dinner). The postprandial increase in blood P concentration in individuals receiving late dinner shifted the gradual increase of serum P concentration to a later time point and resulted in a late onset of serum P concentration apex in the morning compared with individuals receiving early dinner.^28^

Postprandial increases in blood P concentration were reported in cats, and have been attributed to dietary Ca:P, source of P, and P intake.^10^, ^29^, ^30^ In a previous study,^10^ apparently healthy, intact male cats were offered complete and balanced dry diets with similar P concentration and Ca:P for 2‐hour ad libitum feeding before blood sampling. Apparent postprandial increases in blood P concentration were seen in cats offered diets formulated with predominantly inorganic, relatively soluble P.^10^ In these cats, blood P concentration increased by approximately 80% by 2 hours after feeding and then maintained a plateau for 2 hours.^10^ Such an increase in blood P concentration also was seen in cats offered a diet formulated with inorganic, relatively soluble P in another study.^29^ Despite the similar inclusion rate of inorganic, relatively soluble P (64% vs. 73%), compared with the diet offered in the other study,^10^ the diet offered in former study^29^ had a reverse Ca:P and higher total P per 1000 kcal. In addition, regardless of differences in diets offered in these studies (eg, manufacturing process and total dietary fiber content), the absolute amounts of P consumed by cats in these studies were significantly different because of the feeding practice, ad libitum feeding,^10^ compared with food matching 50% of metabolic energy requirement before blood sampling.^29^, ^30^ These differences might affect the interpretation of these data, but clearly, dietary P intake seems to play an important role in the postprandial increase in blood P concentration.

Our study was carefully designed to minimize the impact of these factors. Although the 5% postprandial increase in serum P concentration could not be attributed to dietary intake from a diet predominantly formulated with organic, relatively insoluble P at 09:00 with a high level of certainty because of the limited number of blood samples obtained at 10:00 in our study, an increase in serum P concentration from the nadir around 23:00 independent of dietary intake supports the existence of diurnal variation in serum P concentration in cats.

It was speculated that the diurnal variations in blood ionized Ca, calcidiol, and PTH concentrations play important roles in the circadian rhythm of blood P concentration. Previously, diurnal variation of blood P concentration has been paired with an overall inverted change in blood Ca concentration in humans.^5^, ^6^, ^7^, ^9^ Clinically, the inverse relationship was also evident in some cats with CKD. The diurnal variations of serum ionized Ca concentration were not significant in our study, but a non‐linear relationship between serum ionized Ca and P concentration changes was evident. Such a relationship could not be fully explained by the well‐documented Ca and P metabolism. Although the recently documented calciprotein particles may play a role in this relationship,^31^ further investigations are warranted to determine its mechanism.

Vitamin D in its active form (calcitriol; 1,25‐dihydroxy‐vitamin D) modulates bone mineral metabolism and likely influences the circadian rhythm of blood P concentration. Vitamin D directly and indirectly decreases the concentration of PTH, which contributes to the increased blood P concentration. Conversely, vitamin D can increase blood fibroblast growth factor 23 (FGF 23) concentration and cause subsequent phosphaturia to decrease serum P concentration.^32^ The blood concentration of vitamin D fluctuates during 24‐hour blood collection in humans and has no specific correlation with changes in blood P and ionized Ca concentrations.^33^ Similarly, this feedback system seems not to be responsible for the circadian rhythm of serum P concentration in our study. Interestingly, serum vitamin D concentrations were below the reference range at baseline in 5 cats, and remained low throughout the 24‐hour sampling in 2 of these 5 cats. Inadequate intake is considered unlikely because the cats were fed a diet formulated with cholecalciferol, a highly bioavailable form of vitamin D in cats,^34^ in a concentration exceeding AAFCO^13^ and NRC^18^ nutrient guidelines for adult cats. In addition, these cats' daily calorie intake approximated their calculated energy requirements, and they overall maintained weight stability with ideal BCS throughout the study. Vitamin D undergoes various biochemical modifications to achieve bioactivity or become inactivated. Epimerization results in the documented vitamin D epimers in humans and cats.^35^, ^36^ Given the abundance of vitamin D epimer in cats,^36^ feline blood vitamin D analysis should be interpreted with caution because vitamin D epimers may result in the underestimation of vitamin D using various diagnostic tools, including those based on radioimmunoassay methods.^35^ Consequently, chromatography methods (such as high‐performance liquid chromatography) are required to differentiate epimers from parent compounds of vitamin D. Regardless, a thorough nutritional assessment, consisting of medical and diet evaluations, should be performed in every patient to guide the formulation of diagnostic and therapeutic plans.

In our study, PTH concentrations did not fit into a circadian rhythm model and did not seem to contribute to the rhythm of serum P concentration. Although increase in serum P concentration was attributed to the blunted PTH response and subsequently decreased urinary P excretion in 1 study,^28^ the timing of blood and urine sampling made the finding difficult to interpret. Because of the overall parallel courses of blood PTH and P concentration changes, PTH is unlikely to be associated with the diurnal variation of blood P concentrations in humans and dogs.^5^, ^21^, ^28^, ^33^ We did not assess the urinary excretion of P, and additional investigations are warranted to further characterize this relationship. Regardless, variably increased serum PTH concentration was documented in 1 cat throughout the 24‐hour blood sampling. Overestimation of PTH because of the specificity of antibodies used in various immunoassay methods was reported in humans.^37^, ^38^ In addition, the methodology used to establish reference ranges of certain diagnostic variables also may impact data interpretation.^39^, ^40^ Therefore, assessing the trends of certain analytes should be prioritized over solely assessing these analytes based on reference ranges to provide a better assessment, which emphasizes the value of our study examining the diurnal variation of P and pertinent metabolites.

Along with PTH, FGF 23, secreted by osteocytes, inhibits P reabsorption and has been suspected to contribute to decreasing blood P concentrations in the rhythm. The importance of FGF 23 to diurnal variation of blood P concentration was questionable in humans^28^, ^33^, ^41^ because the change in FGF 23 concentrations and urinary P excretion did not fully correlate with the variation of serum P concentration.^41^ However, at the time our study was conducted, no validated assays for FGF 23 were available. The lack of FGF 23 analysis for such technical reasons is a limitation of our study, and prohibited adequate assessment of such correlation in cats.

Although 3 of the 6 cats in our study had SDMA concentrations above the reference range, they were assessed to be clinically healthy with normal urine concentrating ability and normal serum creatinine and P concentrations. These cats may serve as a good representation of non‐azotemic cats evaluated in the clinical setting. Considering the previously reported discrepancies between humans with and without kidney disease,^33^, ^41^ the diurnal variation of blood P concentration may be more prominent in biochemically and clinically healthy cats compared with cats with CKD. Given the role control of serum P concentration plays in the management of CKD in cats, studies defining the circadian rhythm of serum P concentration in cats with various stages and substages of kidney disease can be considered.

Our study had some limitations. The small sample size of the selected cat population (adult, intact, male cats) may undermine statistical power, especially when not all variables could be assessed at each time point. However, given the statistical analysis applied, we were able to characterize the circadian rhythm of serum P concentration and determine the effect of pertinent metabolites on the rhythm. Second, our study was undertaken in a single 24‐hour period. It would be valuable to repeat the sampling period to confirm the findings as well as to modify the feeding schedule and diet to determine the effects of meals and of different P amounts and sources. Finally, our study was performed under controlled environmental conditions, including meal timing, to accurately observe the variation, which may decrease its generalizability and application to clinical scenarios. Nevertheless, our study provides valuable data to complement existing knowledge of P metabolism in cats.

In conclusion, we demonstrated the diurnal variation in serum P concentration and the association between serum P and ionized Ca concentration changes. Clinicians should assess the results of blood sampling accounting for the expected diurnal variation of serum P concentration as well as the impact of dietary P intake, and evaluate serum P concentration along with ionized Ca status. Interpreting veterinary guidelines for the treatment and monitoring of patients with CKD (such as authoritative consensus statements) should account for the circadian rhythm of relevant blood metabolites.

## CONFLICT OF INTEREST DECLARATION

Chih‐Fan Chiang has attended continuing education events sponsored by Royal Canin and Hill's Pet Nutrition. He was awarded a Hill's Pet Nutrition Resident Clinical Study Grant. He participated as a speaker or attendee in continuing education events hosted or organized by the Taiwan Academy of Veterinary Internal Medicine, Hill's Pet Nutrition Taiwan, and Royal Canin Taiwan. Jonathan Stockman has attended continuing education events sponsored by Nestle Purina and participated as a speaker in events sponsored by Hill's Pet Nutrition. He received research support from Royal Canin and Hill's Pet Nutrition. He is a paid consultant for Petco LLC and was a consultant for Mars Petcare until 2023. Jennifer Larsen is an investigator in clinical trials and other research partly or fully sponsored by Royal Canin, Nature's Variety Instinct, and Nestle Purina PetCare. She develops educational materials for Mark Morris Institute and HealthyPet Magazine, and is an advisory panel member for Purina Institute. She participated in continuing education events, as a speaker or attendee, sponsored or organized by Royal Canin, Nestle Purina PetCare, Nature's Variety Instinct, and Hill's Pet Nutrition. Andrea Fascetti has advised Synergy Food Ingredients and Clorox. She collaborated with and received research and training grants from Nutro and the United States Federal Drug Administration. She has been an event attendee and received remuneration for lectures, or as an advisor on behalf of Nestlé Purina PetCare, Purina Institute, Mars Petcare, Pet Food Institute, and Mark Morris Institute. Raphael Vanderstichel declares no conflict of interest.

## OFF‐LABEL ANTIMICROBIAL DECLARATION

Authors declare no off‐label use of antimicrobials.

## INSTITUTIONAL ANIMAL CARE AND USE COMMITTEE (IACUC) OR OTHER APPROVAL DECLARATION

Approved by the IACUC at the University of California, Davis, #22288.

## HUMAN ETHICS APPROVAL DECLARATION

Authors declare human ethics approval was not needed for this study.

## Supporting information


**Data S1.** Figures.

**Data S2.** Tables.
