# Supplementary material for: Diurnal variation of serum phosphorus concentrations in intact male adult domestic cats
Source: J Vet Intern Med. 2024 Sep 26;38(6):3153–61. doi: 10.1111/jvim.17202 (PMC11586537; doi:10.1111/jvim.17202)
Supplement: Supplementary file 1 — Data S1. Figures. [file JVIM-38-3153-s001.docx]

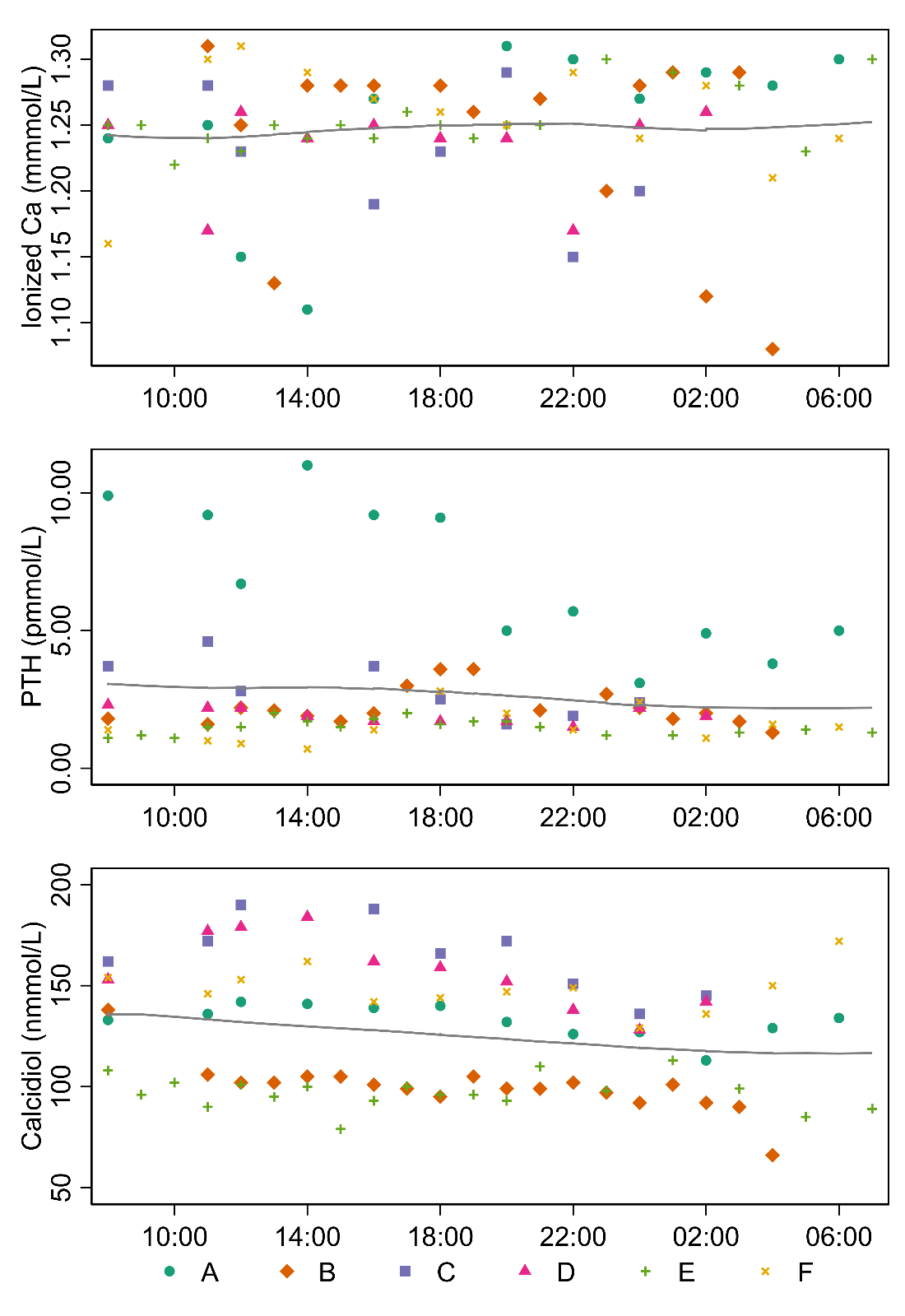


Supplemental Figure 1. Serum ionized Ca (Top; n=76), PTH (Middle; n=78), and calcidiol (Bottom; n=81) concentrations obtained during 24-hour serial blood sampling in six cats (A, B, C, D, E, and F), starting at 08:00. The horizontal grey lines represent locally-weighted scatterplot smoothed (lowess) estimates, with the default bandwidth parameter set at 0.8. No significant temporal predictors were found for these serum concentrations with linear mixed-effect models, using sine and cosine functions and setting cats as a random effect.
