# Supplementary material for: Diurnal variation of serum phosphorus concentrations in intact male adult domestic cats
Source: J Vet Intern Med. 2024 Sep 26;38(6):3153–61. doi: 10.1111/jvim.17202 (PMC11586537; doi:10.1111/jvim.17202)
Supplement: Supplementary file 2 — Data S2. Tables. [file JVIM-38-3153-s002.docx]

Supplemental Table 1: Baseline lab work on Day -15. RR, reference range; Me, median; Q1, the first quantile; Q3, the third quantile. Hyphens represent data non-applicable.

|  | cat | RR | A | B | C | D | E | F | mean | min | Q1 | median | Q3 | max |
| --- | --- | --- | --- | --- | --- | --- | --- | --- | --- | --- | --- | --- | --- | --- |
| Items | Unit |  |  |  |  |  |  |  |  |  |  |  |  |  |
| ANION GAP | mmol/L | 13-27 | 23 | 19 | 21 | 27 | 20 | 27 | 22.83 | 19 | 20.3 | 22.0 | 26.0 | 27 |
| SODIUM | mmol/L | 151-158 | 153 | 151 | 151 | 155 | 152 | 155 | 152.83 | 151 | 151.3 | 152.5 | 154.5 | 155 |
| POTASSIUM | mmol/L | 3.6-4.9 | 4.4 | 4.1 | 4.3 | 4 | 4.3 | 4.3 | 4.23 | 4 | 4.2 | 4.3 | 4.3 | 4.4 |
| CHLORIDE | mmol/L | 117-126 | 115 | 118 | 115 | 116 | 117 | 114 | 115.83 | 114 | 115.0 | 115.5 | 116.8 | 118 |
| BICARBONATE | mmol/L | 15-21 | 20 | 18 | 20 | 16 | 19 | 19 | 18.67 | 16 | 18.3 | 19.0 | 19.8 | 20 |
| PHOSPHORUS | mg/dL | 3.2-6.3 | 4.9 | 3.6 | 4.5 | 3.8 | 5.1 | 5.7 | 4.60 | 3.6 | 4.0 | 4.7 | 5.1 | 5.7 |
| CALCIUM | mg/dL | 9.0-10.9 | 10 | 9.5 | 9.7 | 9.9 | 9.5 | 10.2 | 9.80 | 9.5 | 9.6 | 9.8 | 10.0 | 10.2 |
| BUN | mg/dL | 18-33 | 33 | 25 | 20 | 21 | 25 | 27 | 25.17 | 20 | 22.0 | 25.0 | 26.5 | 33 |
| CREATININE | mg/dL | 1.1-2.2 | 1.3 | 1.7 | 1.1 | 1.2 | 1.1 | 1.5 | 1.32 | 1.1 | 1.1 | 1.3 | 1.5 | 1.7 |
| GLUCOSE | mg/dL | 63-118 | 85 | 107 | 113 | 86 | 149 | 90 | 105.00 | 85 | 87.0 | 98.5 | 111.5 | 149 |
| TP | g/dL | 6.6-8.4 | 7.3 | 6.1 | 6.1 | 7 | 6 | 8.1 | 6.77 | 6 | 6.1 | 6.6 | 7.2 | 8.1 |
| ALBUMIN | g/dL | 2.2-4.6 | 4.1 | 3.7 | 3.6 | 4.4 | 3.7 | 4 | 3.92 | 3.6 | 3.7 | 3.9 | 4.1 | 4.4 |
| GLOBULIN | g/dL | 2.8-5.4 | 3.2 | 2.4 | 2.5 | 2.6 | 2.3 | 4.1 | 2.85 | 2.3 | 2.4 | 2.6 | 3.1 | 4.1 |
| ALT | IU/L | 27-101 | 143 | 53 | 56 | 94 | 33 | 88 | 77.83 | 33 | 53.8 | 72.0 | 92.5 | 143 |
| AST | IU/L | 17-58 | 52 | 44 | 32 | 36 | 26 | 44 | 39.00 | 26 | 33.0 | 40.0 | 44.0 | 52 |
| ALP | IU/L | 14-71 | 72 | 40 | 31 | 46 | 23 | 32 | 40.67 | 23 | 31.3 | 36.0 | 44.5 | 72 |
| GGT | IU/L | 0-4 | <3 | <3 | <3 | <3 | <3 | <3 | - | - | - | - | - | - |
| CHOLESTEROL | mg/dL | 89-258 | 144 | 100 | 110 | 81 | 107 | 117 | 109.83 | 81 | 101.8 | 108.5 | 115.3 | 144 |
| T. BILIRUBIN | mg/dL | 0.0-0.2 | <0.2 | <0.2 | <0.2 | <0.2 | <0.2 | <0.2 | - | - | - | - | - | - |
| MAGNESIUM | mg/dL | 1.5-2.5 | 2.2 | 2.1 | 2 | 2.1 | 2.2 | 2.6 | 2.20 | 2 | 2.1 | 2.2 | 2.2 | 2.6 |
| TOTAL T4 | mcg/dL | 1.1-3.3 | 1.9 | 2.2 | 1.9 | 1.7 | 1.2 | 1.5 | 1.73 | 1.2 | 1.6 | 1.8 | 1.9 | 2.2 |
| SDMA | mcg/dL | 0-14 | 16 | 17 | 13 | 13 | 14 | 15 | 14.67 | 13 | 13.3 | 14.5 | 15.8 | 17 |
| Vitamin D Profile |  |  |  |  |  |  |  |  |  |  |  |  |  |  |
| PTH | pmol/L | 0.7-3.4 | 4.1 | 6.6 | 2.7 | 0.9 | 0.9 | 4.9 | 3.35 | 0.9 | 1.4 | 3.4 | 4.7 | 6.6 |
| iCa | mmol/L | 1.0-1.4 | 1.25 | 1.24 | 1.26 | 1.23 | 1.26 | 1.22 | 1.24 | 1.22 | 1.2 | 1.2 | 1.3 | 1.26 |
| 25-OH-D | nmol/L | 127-335 | 125 | 102 | 115 | 165 | 95 | 121 | 120.50 | 95 | 105.3 | 118.0 | 124.0 | 165 |
| UA (CYSTO) |  |  |  | N/A |  |  |  |  |  |  |  |  |  |  |
| COLOR | - | - | Yellow | - | Yellow | Yellow | Yellow | Yellow | - | - | - | - | - | - |
| CLARITY | - | - | Cloudy | - | Opaque | Opaque | Cloudy | Cloudy | - | - | - | - | - | - |
| USG | - | - | 1.041 | - | 1.047 | 1.052 | 1.045 | 1.045 | - | - | - | - | - | - |
| PH | - | - | 8 | - | 7 | 7 | 6.5 | 7 | - | - | - | - | - | - |
| PROTEIN | mg/dL | neg | 100 | - | 100 | 100 | 100 | 100 | - | - | - | - | - | - |
| GLUCOSE | mg/dL | neg | neg | - | neg | neg | neg | neg | - | - | - | - | - | - |
| KETONES | mg/dL | neg | 5 | - | 5 | 5 | 15 | 5 | - | - | - | - | - | - |
| BILIRUBIN | mg/dL | neg | neg | - | neg | neg | neg | neg | - | - | - | - | - | - |
| HEMOPROTEIN | ERY/uL | neg | neg | - | neg | 250 | 250 | neg | - | - | - | - | - | - |
| WBC | /HPF | neg | 0-3 | - | 0-3 | 0-3 | 0-3 | 0-3 | - | - | - | - | - | - |
| RBC | /HPF | neg | None | - | Rare | 25-50 | 50-100 | Rare | - | - | - | - | - | - |
| CRYSTALS | /HPF | neg | Many | - | Few | Rare | Mod | Few | - | - | - | - | - | - |
| BACTERIA | /HPF | neg | None | - | None | None | None | None | - | - | - | - | - | - |

Supplemental Table 2: The serum phosphorus concentration (mg/dL) at each time point of 6 cats (A, B, C, D, E, and F) on Day 0 undergoing the 24-hour serial blood sampling. Me, median; Q1, the first quantile; Q3, the third quantile. Hyphens represent data non-applicable.

| Time | A | B | C | D | E | F | Me | Q1 | Q3 |
| --- | --- | --- | --- | --- | --- | --- | --- | --- | --- |
| 0800 | 4.6 | 6.2 | 5.0 | 5.3 | 6.0 | 6.4 | 5.65 | 5.08 | 6.15 |
| 0900 | - | - | - | - | 6.3 | - | 6.30 | 6.30 | 6.30 |
| 1000 | - | - | - | - | 6.9 | - | 6.90 | 6.90 | 6.90 |
| 1100 | 5.2 | 4.2 | 4.9 | 5.4 | 6.7 | 5.0 | 5.10 | 4.93 | 5.35 |
| 1200 | 4.2 | 4.2 | 4.9 | 5.2 | 6.2 | 5.3 | 5.05 | 4.38 | 5.28 |
| 1300 | - | 3.7 | - | - | 5.8 | - | 4.75 | 4.23 | 5.28 |
| 1400 | 4.1 | 3.4 | 4.5 | 5.5 | 5.4 | 6.0 | 4.95 | 4.20 | 5.48 |
| 1500 | - | 3.9 | - | - | 6.0 | - | 4.95 | 4.43 | 5.48 |
| 1600 | 4.0 | 4.3 | 4.6 | 4.9 | 5.6 | 6.0 | 4.75 | 4.38 | 5.43 |
| 1700 | - | 4.8 | - | - | 6.2 | - | 5.50 | 5.15 | 5.85 |
| 1800 | 4.1 | 3.9 | 4.0 | 5.0 | 5.5 | 5.3 | 4.55 | 4.03 | 5.23 |
| 1900 | - | 3.6 | - | - | 5.6 | - | 4.60 | 4.10 | 5.10 |
| 2000 | 3.9 | 3.2 | 4.3 | 4.7 | 5.7 | 4.6 | 4.45 | 4.00 | 4.68 |
| 2100 | - | 3.2 | - | - | 3.9 | - | 3.55 | 3.38 | 3.73 |
| 2200 | 3.9 | 3.6 | 4.7 | 4.4 | - | 4.4 | 4.40 | 3.90 | 4.40 |
| 2300 | - | 3.6 | - | - | 3.2 | - | 3.40 | 3.30 | 3.50 |
| 0000 | 4.1 | 3.4 | 4.5 | 4.9 | - | 4.7 | 4.50 | 4.10 | 4.70 |
| 0100 | - | 3.6 | - | - | 3.7 | - | 3.65 | 3.63 | 3.68 |
| 0200 | 4.1 | 3.5 | 4.8 | 4.7 | - | 4.4 | 4.40 | 4.10 | 4.70 |
| 0300 | - | 3.6 | - | - | 4.2 | - | 3.90 | 3.75 | 4.05 |
| 0400 | 4.2 | 2.5 | - | - | - | 4.7 | 4.20 | 3.35 | 4.45 |
| 0500 | - | - | - | - | 4.8 | - | 4.80 | 4.80 | 4.80 |
| 0600 | 4.0 | - | - | - | - | 4.9 | 4.45 | 4.23 | 4.68 |
| 0700 | - | - | - | - | 3.8 | - | 3.80 | 3.80 | 3.80 |

Supplemental Table 3: The serum ionized Ca concentration (mmol/L) at each time point of 6 cats (A, B, C, D, E, and F) on Day 0 undergoing the 24-hour serial blood sampling. Me, median; Q1, the first quantile; Q3, the third quantile. Hyphens represent data non-applicable.

| Time | A | B | C | D | E | F | Me | Q1 | Q3 |
| --- | --- | --- | --- | --- | --- | --- | --- | --- | --- |
| 08:00 | 1.24 | - | 1.28 | 1.25 | 1.25 | 1.16 | 1.250 | 1.240 | 1.250 |
| 09:00 | - | - | - | - | 1.25 | - | 1.250 | 1.250 | 1.250 |
| 10:00 | - | - | - | - | 1.22 | - | 1.220 | 1.220 | 1.220 |
| 11:00 | 1.25 | 1.31 | 1.28 | 1.17 | 1.24 | 1.30 | 1.265 | 1.243 | 1.295 |
| 12:00 | 1.15 | 1.25 | 1.23 | 1.26 | 1.23 | 1.31 | 1.240 | 1.230 | 1.258 |
| 13:00 | - | 1.13 | - | - | 1.25 | - | 1.190 | 1.160 | 1.220 |
| 14:00 | 1.11 | 1.28 | - | 1.24 | 1.24 | 1.29 | 1.240 | 1.240 | 1.280 |
| 15:00 | - | 1.28 | - | - | 1.25 | - | 1.265 | 1.258 | 1.273 |
| 16:00 | 1.27 | 1.28 | 1.19 | 1.25 | 1.24 | 1.27 | 1.260 | 1.243 | 1.270 |
| 17:00 | - | - | - | - | 1.26 | - | 1.260 | 1.260 | 1.260 |
| 18:00 | 1.28 | 1.28 | 1.23 | 1.24 | 1.25 | 1.26 | 1.255 | 1.243 | 1.275 |
| 19:00 | - | 1.26 | - | - | 1.24 | - | 1.250 | 1.245 | 1.255 |
| 20:00 | 1.31 | - | 1.29 | 1.24 | 1.25 | 1.25 | 1.250 | 1.250 | 1.290 |
| 21:00 | - | 1.27 | - | - | 1.25 | - | 1.260 | 1.255 | 1.265 |
| 22:00 | 1.30 | - | 1.15 | 1.17 | - | 1.29 | 1.230 | 1.165 | 1.293 |
| 23:00 | - | 1.20 | - | - | 1.30 | - | 1.250 | 1.225 | 1.275 |
| 00:00 | 1.27 | 1.28 | 1.20 | 1.25 | - | 1.24 | 1.250 | 1.240 | 1.270 |
| 01:00 | - | 1.29 | - | - | 1.29 | - | 1.290 | 1.290 | 1.290 |
| 02:00 | 1.29 | 1.12 | - | 1.26 | - | 1.28 | 1.270 | 1.225 | 1.283 |
| 03:00 | - | 1.29 | - | - | 1.28 | - | 1.285 | 1.283 | 1.288 |
| 04:00 | 1.28 | 1.08 | - | - | - | 1.21 | 1.210 | 1.145 | 1.245 |
| 05:00 | - | - | - | - | 1.23 | - | 1.230 | 1.230 | 1.230 |
| 06:00 | 1.30 | - | - | - | - | 1.24 | 1.270 | 1.255 | 1.285 |
| 07:00 | - | - | - | - | 1.30 | - | 1.300 | 1.300 | 1.300 |

Supplemental Table 4. The serum PTH concentration (pmol/L) at each time point of 6 cats (A, B, C, D, E, and F) on Day 0 undergoing the 24-hour serial blood sampling. Me, median; Q1, the first quantile; Q3, the third quantile. Hyphens represent data non-applicable.

| Time | A | B | C | D | E | F | Me | Q1 | Q3 |
| --- | --- | --- | --- | --- | --- | --- | --- | --- | --- |
| 08:00 | 9.9 | 1.8 | 3.7 | 2.3 | 1.1 | 1.4 | 2.05 | 1.50 | 3.35 |
| 09:00 | - | - | - | - | 1.2 | - | 1.20 | 1.20 | 1.20 |
| 10:00 | - | - | - | - | 1.1 | - | 1.10 | 1.10 | 1.10 |
| 11:00 | 9.2 | 1.6 | 4.6 | 2.2 | 1.5 | 1.0 | 1.90 | 1.53 | 4.00 |
| 12:00 | 6.7 | 2.2 | 2.8 | 2.2 | 1.5 | 0.9 | 2.20 | 1.68 | 2.65 |
| 13:00 | - | 2.1 | - | - | 2.0 | - | 2.05 | 2.03 | 2.08 |
| 14:00 | 11.0 | 1.9 | - | 1.9 | 1.7 | 0.7 | 1.90 | 1.70 | 1.90 |
| 15:00 | - | 1.7 | - | - | 1.5 | - | 1.60 | 1.55 | 1.65 |
| 16:00 | 9.2 | 2.0 | 3.7 | 1.7 | 1.8 | 1.4 | 1.90 | 1.73 | 3.28 |
| 17:00 | - | 3.0 | - | - | 2.0 | - | 2.50 | 2.25 | 2.75 |
| 18:00 | 9.1 | 3.6 | 2.5 | 1.7 | 1.6 | 2.8 | 2.65 | 1.90 | 3.40 |
| 19:00 | - | 3.6 | - | - | 1.7 | - | 2.65 | 2.18 | 3.13 |
| 20:00 | 5.0 | - | 1.6 | 1.7 | 1.7 | 2.0 | 1.70 | 1.70 | 2.00 |
| 21:00 | - | 2.1 | - | - | 1.5 | - | 1.80 | 1.65 | 1.95 |
| 22:00 | 5.7 | - | 1.9 | 1.5 | - | 1.4 | 1.70 | 1.48 | 2.85 |
| 23:00 | - | 2.7 | - | - | 1.2 | - | 1.95 | 1.58 | 2.33 |
| 00:00 | 3.1 | 2.2 | 2.4 | 2.2 | - | 2.4 | 2.40 | 2.20 | 2.40 |
| 01:00 | - | 1.8 | - | - | 1.2 | - | 1.50 | 1.35 | 1.65 |
| 02:00 | 4.9 | 2.0 | - | 1.9 | - | 1.1 | 1.95 | 1.70 | 2.73 |
| 03:00 | - | 1.7 | - | - | 1.3 | - | 1.50 | 1.40 | 1.60 |
| 04:00 | 3.8 | 1.3 | - | - | - | 1.6 | 1.60 | 1.45 | 2.70 |
| 05:00 | - | - | - | - | 1.4 | - | 1.40 | 1.40 | 1.40 |
| 06:00 | 5.0 | - | - | - | - | 1.5 | 3.25 | 2.38 | 4.13 |
| 07:00 | - | - | - | - | 1.3 | - | 1.30 | 1.30 | 1.30 |

Supplemental Table 5: The serum calcidiol concentration (nmol/L) at each time point of 6 cats (A, B, C, D, E, and F) on Day 0 undergoing the 24-hour serial blood sampling. Me, median; Q1, the first quantile; Q3, the third quantile. Hyphens represent data non-applicable.

| Time | A | B | C | D | E | F | Me | Q1 | Q3 |
| --- | --- | --- | --- | --- | --- | --- | --- | --- | --- |
| 08:00 | 133 | 138 | 162 | 153 | 108 | 154 | 145.5 | 134.3 | 153.8 |
| 09:00 | - | - | - | - | 96 | - | 96.0 | 96.0 | 96.0 |
| 10:00 | - | - | - | - | 102 | - | 102.0 | 102.0 | 102.0 |
| 11:00 | 136 | 106 | 172 | 177 | 90 | 146 | 141.0 | 113.5 | 165.5 |
| 12:00 | 142 | 102 | 190 | 179 | 101 | 153 | 147.5 | 112.0 | 172.5 |
| 13:00 | - | 102 | - | - | 95 | - | 98.5 | 96.8 | 100.3 |
| 14:00 | 141 | 105 | - | 184 | 100 | 162 | 141.0 | 105.0 | 162.0 |
| 15:00 | - | 105 | - | - | 79 | - | 92.0 | 85.5 | 98.5 |
| 16:00 | 139 | 101 | 188 | 162 | 93 | 142 | 140.5 | 110.5 | 157.0 |
| 17:00 | - | 99 | - | - | 100 | - | 99.5 | 99.3 | 99.8 |
| 18:00 | 140 | 95 | 166 | 159 | 96 | 144 | 142.0 | 107.0 | 155.3 |
| 19:00 | - | 105 | - | - | 96 | - | 100.5 | 98.3 | 102.8 |
| 20:00 | 132 | 99 | 172 | 152 | 93 | 147 | 139.5 | 107.3 | 150.8 |
| 21:00 | - | 99 | - | - | 110 | - | 104.5 | 101.8 | 107.3 |
| 22:00 | 126 | 102 | 151 | 138 | - | 149 | 138.0 | 126.0 | 149.0 |
| 23:00 | - | 97 | - | - | 98 | - | 97.5 | 97.3 | 97.8 |
| 00:00 | 127 | 92 | 136 | 128 | - | 129 | 128.0 | 127.0 | 129.0 |
| 01:00 | - | 101 | - | - | 113 | - | 107.0 | 104.0 | 110.0 |
| 02:00 | 113 | 92 | 145 | 142 | - | 136 | 136.0 | 113.0 | 142.0 |
| 03:00 | - | 90 | - | - | 99 | - | 94.5 | 92.3 | 96.8 |
| 04:00 | 129 | 66 | - | - | - | 150 | 129.0 | 97.5 | 139.5 |
| 05:00 | - | - | - | - | 85 | - | 85.0 | 85.0 | 85.0 |
| 06:00 | 134 | - | - | - | - | 172 | 153.0 | 143.5 | 162.5 |
| 07:00 | - | - | - | - | 89 | - | 89.0 | 89.0 | 89.0 |
